# Mechanisms underlying TNFα‐induced enhancement of force generation in airway smooth muscle

**DOI:** 10.14814/phy2.14220

**Published:** 2019-09-11

**Authors:** Gary C. Sieck, Murat Dogan, Han Young‐Soo, Sara Osorio Valencia, Philippe Delmotte

**Affiliations:** ^1^ Department of Physiology and Biomedical Engineering Mayo Clinic Rochester Minnesota

**Keywords:** Actin polymerization, inflammation, myosin heavy chain, myosin light chain phosphorylation

## Abstract

Airway diseases such as asthma are triggered by inflammation and mediated by proinflammatory cytokines such as tumor necrosis factor alpha (TNFα). Our goal was to systematically examine the potential mechanisms underlying the effect of TNFα on airway smooth muscle (ASM) contractility. Porcine ASM strips were incubated for 24 h with and without TNFα. Exposure to TNFα increased maximum ASM force in response to acetylcholine (Ach), with an increase in ACh sensitivity (hyperreactivity), as reflected by a leftward shift in the dose–response curve (EC_50_). At the EC_50_, the [Ca^2+^]_cyt_ response to ACh was similar between TNFα and control ASM, while force increased; thus, Ca^2+^ sensitivity appeared to increase. Exposure to TNFα increased the basal level of regulatory myosin light chain (rMLC) phosphorylation in ASM; however, the ACh‐dependent increase in rMLC phosphorylation was blunted by TNFα with no difference in the extent of rMLC phosphorylation at the EC_50_ ACh concentration. In TNFα‐treated ASM, total actin and myosin heavy chain concentrations increased. TNFα exposure also enhanced the ACh‐dependent polymerization of G‐ to F‐actin. The results of this study confirm TNFα‐induced hyperreactivity to ACh in porcine ASM. We conclude that the TNFα‐induced increase in ASM force, cannot be attributed to an enhanced [Ca^2+^]_cyt_ response or to an increase in rMLC phosphorylation. Instead, TNFα increases Ca^2+^ sensitivity of ASM force generation due to increased contractile protein content (greater number of contractile units) and enhanced cytoskeletal remodeling (actin polymerization) resulting in increased tethering of contractile elements to the cortical cytoskeleton and force translation to the extracellular matrix.

## Introduction

A methacholine (MCh) challenge test is usually used to help diagnose asthma, which is associated with an increase in airway smooth muscle (ASM) sensitivity to muscarinic stimulation (hypersensitivity, shift in the EC_50_) and/or with increased ASM force response to muscarinic stimulation (hypercontractility). ASM hyperreactivity is also used to characterize asthmatic ASM but is not well defined. It is not always clear whether ASM hyperreactivity refers to hypersensitivity and/or hypercontractility to muscarinic stimulation. Airway inflammation is a common feature of chronic lung diseases such as asthma and is associated with increased ASM contractility (Martin et al., 2000; Prakash, 2013; West et al., 2013; Wright et al., 2013). It is likely that proinflammatory cytokines such as tumor necrosis factor alpha (TNF) mediate the influence of inflammation on ASM contractility. Previous studies have shown that exposure to TNFα affects ASM force sensitivity to muscarinic stimulation (Kao et al., 1999; Nakatani et al., 2000; Sakai et al., 2004a,2004b). Acetylcholine (ACh) acting through muscarinic receptors is a major agonist of ASM contraction. Muscarinic stimulation involves multiple steps initiated by an ACh‐induced increase in cytosolic Ca^2+^ concentration ([Ca^2+^]_cyt_), followed by Ca^2+^ binding to calmodulin (CaM), Ca^2+^/CaM activation of myosin light chain kinase, phosphorylation of a regulatory myosin light chain (rMLC), myosin heavy chain (MyHC) binding to actin (cross‐bridge formation), tethering of actin filaments to the ASM membrane and force generation against an external load (Murphy et al., 1984; Murphy, 1989; Gunst and Tang, 2000; Sieck et al., 2001; Murphy and Rembold, 2005; Sieck and Gransee, 2012).

Previous studies have shown that exposure to TNFα enhances ACh‐induced ASM force generation (Amrani and Panettieri, 1998; Sathish et al., 2011b; Makwana et al., 2012; Amrani, 2014), which has been primarily attributed to an increase in [Ca^2+^]_cyt_ (Sathish et al., 2008; Sathish et al., 2009; Sathish et al., 2011a; Delmotte et al., 2012; Sathish et al., 2014), and/or an increase in Ca^2+^ sensitivity (Croxton et al., 1998; Parris et al., 1999; Nakatani et al., 2000; Hunter et al., 2003). However, the same concentration of ACh was used in these studies to compare responses in both control and TNF‐exposed groups, essentially ignoring any effect of TNFα exposure on ASM force sensitivity to ACh (EC_50_). Surprisingly, no comprehensive study has simultaneously investigated the effect of TNFα exposure on ASM force, [Ca^2+^]_cyt_ responses and Ca^2+^ sensitivity. In a recent study using permeabilized porcine ASM, we found that TNFα increases force generation during maximum Ca^2+^ activation but did not affect Ca^2+^ sensitivity, as reflected by the [Ca^2+^] at which 50% maximum force is generated (pCa_50_), or by the extent of rMLC phosphorylation (Dogan et al., 2017). However, 24‐h TNFα exposure did increase the total actin and MyHC concentrations in ASM. These results suggested that TNFα‐induced enhancement of ASM force generation is primarily due to an increase in the number of contractile elements. However, permeabilization of ASM substantially removes the plasma membrane and thereby might disrupt activation of membrane‐dependent intracellular signaling cascades that affect Ca^2+^ sensitivity or the extent of rMLC phosphorylation.

In the present study, we hypothesized that TNFα‐induced enhancement of ASM force is due to the combined effects of increased sensitivity to muscarinic stimulation, increased [Ca^2+^]_cyt_ response to muscarinic stimulation, increased Ca^2+^ sensitivity of force generation, increased rMLC phosphorylation, increased number of contractile elements, and/or increased tethering of contractile elements to the ASM membrane.

## Material and Methods

### Preparation of porcine ASM strips

Porcine trachea were obtained from a local abattoir and immersed in ice‐cold physiologic saline solution (PSS; composition in mmol/L: 118.9 NaCl, 1.2 MgSO_4_, 1.2 KH_2_PO_4_, 4.7 KCl, 2.5 CaCl_2_, 0.03 EDTA, 5.5 glucose, 25 HEPES). From each trachea, two ASM strips (~0.5 mm wide and 2–4 mm long) were dissected as previously described (Fredberg et al., 1996; Sieck et al., 1998; Jones et al., 1999a,1999b; Sieck et al., 2001; Dogan et al., 2017). One ASM strip was incubated with PSS, and the other was exposed to TNFα (100 ng/mL; Cell Sciences, MA) for 24 h at 24°C (Dogan et al., 2017). This temperature was selected as we have previously noted some rundown in ASM force over a 24‐h period at 37°C.

### Measurement of isometric force response to ACh stimulation

Isometric force generated by ASM strips was measured in response to ACh stimulation as previously described (Jones et al., 1994; Jones et al., 1995; Kuo et al., 2003a). Briefly, ASM strips (control and TNFα‐treated) were mounted horizontally between a force transducer (Danish Myo Technology, Denmark) and a micropositioner (Mitutoyo, Japan; for length adjustment) in a 4‐chamber microvessel system (DMT Myographs System, Denmark) and incubated with PSS (aerated with 95% O_2_ and 5% CO_2_) maintained at 24°C. Muscle length was adjusted such that a passive tension (10 mN) was applied to ASM strips during a 1‐h equilibration period. ASM strips were then stimulated with increasing ACh concentrations (10, 100 nmol/L, 1, 10, 100 μmol/L, and 1 mmol/L) for 5 min at each concentration until a force plateau was reached. Between each ACh concentration, the ASM strips were washed with PSS for 10 min to allow full relaxation before exposure to the next ACh concentration. Isometric force at varying ACh concentrations was recorded using LabChart Pro software (AD Instruments, RRID:SCR_001620), and normalized to cross‐sectional area of the ASM strip (specific force). Maximum specific force (*F*_max_) and the ACh concentration at which 50% *F*_max_ was evoked (EC_50_ – an index of sensitivity to ACh) were determined.

### Simultaneous measurements of [Ca^2+^]_cyt_ and isometric force responses to ACh stimulation

The methods used to simultaneously measure isometric force and [Ca^2+^]_cyt_ responses to ACh stimulation have been previously described (Jones et al., 1994; Jones et al., 1995). Briefly, ASM strips were incubated with PSS containing 5 μmol/L fura‐2 AM for 3 h followed by extensive washing. The ASM strips were then mounted between a force transducer (KG4; Scientific Instruments, Germany) and a micromanipultor (Mitutoyo; to adjust preload to 10 mN) in a 50 μL cuvette contained within a Guth Muscle Research System. After a 1‐h equilibration period, control or TNF‐exposed ASM strips were stimulated with three ACh concentrations: 1 μmol/L, the EC_50_ concentration for each condition (1.3 μmol/L for TNFα‐treated and 2.6 μmol/L for control) and 10 μmol/L, reflecting the steepest portion of the dose–response curve. A high‐pressure mercury lamp (75 W) was used as a light source for fura‐2 excitation, and the light was alternatively filtered at 340 and 380 nm to limit excitation to these wavelengths. The emitted fluorescence was detected at 510 nm. The fluorescence emission induced at 340 and 380 nm excitation (F340/F380) was measured, and [Ca^2+^]_cyt_ was determined using the calibration equation described by Grynkiewicz et al., (1985). Isometric force and [Ca^2+^]_cyt_ responses were digitally recorded in real time (sampling frequency; 100 Hz) and analyzed using LabChart Pro software.

### Measurement of rMLC phosphorylation response to ACh stimulation

Phosphorylation of rMLC was assessed using Phos‐tag^™^ sodium dodecyl sulfate polyacrylamide gel electrophoresis (SDS‐PAGE) gels with Zn^2+^ (Takeya et al., 2008; Kikkawa et al., 2010; Walsh et al., 2011) (Wako Chemicals Inc., Richmond, VA) using a standard western blotting technique (see below). From each trachea, 18 ASM strips (three TNFα‐treated and three control) were stimulated with one of three ACh concentrations: 1 μmol/L, the EC_50_ concentration for each condition (1.3 μmol/L for TNFα or 2.6 μmol/L for control) and 10 μmol/L for 30 sec (previously shown to correspond to peak phosphorylation of rMLC). The strips were then flash‐frozen in 10% trichloroacetic acid (TCA)/10 mmol/L dithiothreitol (DTT) in prechilled acetone. For protein extraction, the strips were washed with 10 mmol/L DTT in acetone to remove TCA. Samples were then minced and ground in 2% SDS, 50 mmol/L DTT, 50 mmol/L Bis‐Tris pH 6.8, 1 tbt Roche EDTA‐free protease inhibitor, 1 tbt Roche PhosStop phosphatase inhibitor, 5% glycerol, and 0.01% Bromophenol Blue. Electrophoresis of the Phos‐tag^™^ gels was performed at 22 mA for 1 h 45 min in running buffer (pH 7.4; 100 mmol/L Tris‐base, 100 mmol/L 3‐morpholinopropane‐1‐sulfonic acid, 0.1% SDS, 5 mmol/L Sodium Bisulfate). The gel was then immersed in transfer buffer (25 mmol/L Tris, 192 mmol/L glycine, 10% methanol [v/v]) containing 10 mmol/L EDTA to remove Zn^2+^ for 30 min and proteins were transferred to polyvinylidene difluoride membrane. The membrane was fixed with 0.5% formaldehyde in PBS for 45 min (Takeya et al., 2008; Kikkawa et al., 2010) and blocked with 5% dry milk in TBST. Using a standard western blotting technique, the membrane was incubated with primary (1:1000 dilution, rabbit polyclonal anti‐rMLC; Santa Cruz Biotechnology Cat# sc‐15370, RRID:AB_2148039) and secondary antibody (1:10,000 dilution, Santa Cruz Biotechnology Cat# sc‐2030, RRID:AB_631747). Unphosphorylated rMLC and phosphorylated rMLC (p‐rMLC) were detected by enhanced chemiluminescence (ECL, SuperSignal West Dura Extended Duration Substrate; Thermo Scientific, Rockford, IL) and imaged on Kodak Image System (Kodak Inc., Rochester, NY) for analysis.

### NM versus SM rMLC phosphorylation

In a separate set of experiments, phosphorylation of nonmuscle (NM) and smooth muscle (SM) rMLC was assessed using two‐dimensional (2D) gel electrophoresis as previous described (Yuen et al., 2009; Han and Brozovich, 2013; Konik et al., 2013). For each trachea (three TNFα‐treated and three control), ASM strips were either unstimulated or stimulated with ACh, the EC50 concentration for each condition (1.3 μmol/L for TNFα or 2.6 μmol/L for control) for 4 min. ASM strips were then homogenized in 2D gel extraction buffer (7 mol/L urea, 2 mol/L thiourea, 4% CHAPS, 1% pH 3–5.6 immobilized pH gradient [IPG] buffer, and Roche EDTA‐free protease inhibitor and Roche PhosStop phosphatase inhibitor). All homogenized samples were cleared of lipids and extraneous salts using the 2D clean up kit (GE Healthcare, Chicago, IL). The acidic halves of the 13‐cm IPG DryStrip gels (pH 4–7 NL) were rehydrated with suitable amounts of sample including rehydration buffer solution (7 mol/L urea, 2 mol/L thiourea, 2% CHAPS, 0.5% pH 3.5–5 IPG buffer, 0.002% bromophenol blue, and 12 μM/mL Destreak Reagent) for at least 10 h in the “face‐down” mode on the Ettan IPG rehydration tray and then resolved by isoelectric focusing (IEF) in the “face‐up” mode on an Ettan IPGphor III (GE Healthcare). Following IEF, the gel strips were equilibrated in equilibration solution (6 mol/L urea, 50 mmol/L Tris–HCl, pH 6.4, 30% glycerol, 2% [w/v] SDS, and 0.002% bromophenol blue), first containing 130 mmol/L DTT for 15 min and then containing 135 mmol/L iodoacetamide for 15 min before undergoing SDS‐PAGE for protein separation by molecular weight using the Bis–Tris buffering system with 12% gels (29:1). Subsequently, resolved 2D SDS‐PAGE gels were silver stained and gels were scanned using a Personal Densitometer SI, and the spots were quantified using ImageQuant TL software. The two spots closest to the anode (spots 1 and 2) represent the phosphorylated and nonphosphorylated NM rMLC and the two spots nearest the cathode (spots 3 and 4) represent the phosphorylated and nonphosphorylated SM rMLC (Yuen et al., 2009). The expression of NM myosin to total myosin is calculated as [(1 + 2)/(1 + 2 + 3 + 4)] × 100%], while the ratio of phosphorylated NM to phosphorylated SM is (1/3) × 100%.

### Measurement of actin and MyHC concentrations in ASM

To measure actin and MyHC concentrations in ASM (control or TNF‐treated), strips were snap‐frozen in liquid nitrogen. For actin measurements, protein was extracted using a radio‐immuno precipitation assay (RIPA, Cell Signaling Technology, Danvers, MA) buffer. Protein extracted from the ASM samples was then separated using a 12% SDS gel (20 µg per well) together with the separation of varying concentrations of known actin standards (AKL99, purified actin protein, rabbit skeletal muscle, Cytoskeleton Inc., Denver, CO) (Dogan et al., 2017). Gels were incubated with a fixing solution composed of 40% ethanol and 10% acetate for 1 h and then incubated with Coomassie Blue stain (Bio‐Safe Coomassie Stain, Bio‐Rad Inc., Hercules, CA) for 1 h. Brightness‐area product (BAP) of each actin band was measured, and actin concentrations of the samples determined by comparison to the standard curve constructed on the basis of the linear relationship between BAP and known actin standards.

In additional adjacent ASM strips from each trachea, protein was extracted using RIPA buffer. Protein extracts (20 µg) and known MyHC standards (M‐3889, purified MyHC protein, rabbit skeletal muscle, Sigma Inc., St. Louis, MO) were separated using a 4–15% SDS gel run at a constant voltage of 40 V for 6 h at 4°C. Gels were stained with Coomassie Blue stain and MyHC concentrations were calculated using the same method as for actin concentrations.

### Measurement of filamentous and globular actin in ASM

The extent of polymerization of globular (G‐) to filamentous (F‐) actin in ASM strips was evaluated using a G‐/F‐actin in vivo assay kit (Cytoskeleton Inc., Denver, CO) as previously described (Jones et al., 1999b; Dogan et al., 2017). From each trachea, 32 ASM strips were dissected (four TNFα‐treated and 4 control × 4 ACh concentrations) to evaluate the effect of ACH stimulation on actin polymerization. The ASM strips were exposed to 0 μmol/L, 1 μmol/L, the EC_50_ concentration for each condition (1.3 μmol/L for TNFα and 2.6 μmol/L for control) and 10 μmol/L for 2 min and then flash‐frozen in liquid nitrogen. Subsequently, the ASM strips were thawed at room temperature and then minced in F‐actin stabilization buffer (composition in mmol/L; 50 PIPES pH 6.9, 50 KCl, 5 MgCl_2_, 5 EGTA, 5% [v/v] glycerol, 0.1% Nonidet P40, 0.1% Triton X‐100, 0.1% Tween 20, and 0.1 % 2‐mercaptoethanol, supplemented with 1 mmol/L ATP and 1% protease inhibitor cocktail). Separation of F‐ and G‐actin was performed using standard western blotting technique with incubations of primary rabbit polyclonal anti‐actin antibody (1:1000 dilution) (Cytoskeleton Cat# AAN01, RRID:AB_10708070) and secondary peroxidase AffiniPure goat anti‐rabbit HRP IgG (1:10 000 dilution) (Jackson ImmunoResearch Labs Cat# 111‐035‐144, RRID:AB_2307391). The actin bands were analyzed with a Kodak Image System (Kodak Inc., CA, USA).

### Statistical analysis

Experiments were performed using porcine tracheas obtained from a total of 36 animals. A total of *n* = 6 animals was used for each set of experiments with ASM strips from the same animal assigned to either TNFα‐treated or control (PSS) groups (paired comparison). Statistical analyses were performed using JMP Pro software (JMP, RRID:SCR_014242). A t‐test or paired t‐test with Bonferroni corrections, and post hoc Turkey test was used when appropriate. For the dose–response data, the Hill equation algorithm (Sigma Plot 12.0, Systat Software, Inc., CA, USA) was used for each individual experiment to determine min, max, EC_50_, and the Hill coefficient. Statistical analysis was conducted on the log‐transformed dose–response curve. All results are presented either as means ± standard deviation or as medians and interquartile range represented as a box‐and‐whisker plot. Differences were considered significant at *P* < 0.05.

## Results

### Effect of TNFα on isometric force

**Figure 1:**
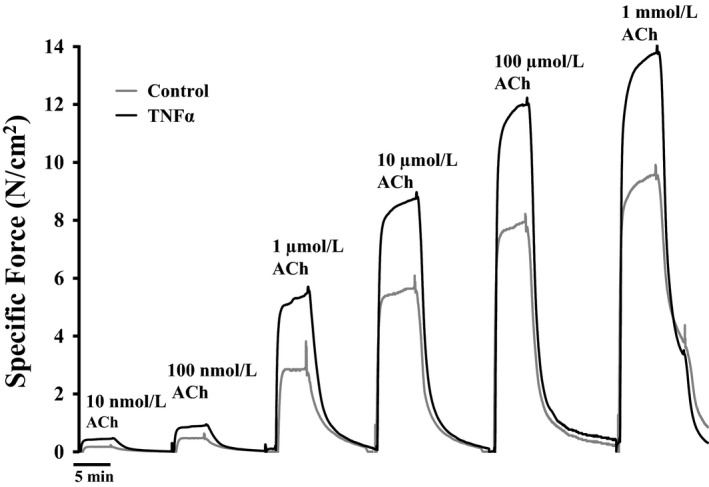
Representative tracings of specific force (force per muscle cross‐sectional area) generated by stimulating with different concentrations of ACh (ranging from 10 nmol/L to 1 mmol/L) in porcine ASM strips. Note that 24‐h exposure to TNFα increased ASM force generation across all ACh concentrations compared to control (*P* < 0.01). ACh, acetylcholine; ASM, airway smooth muscle; TNFα, tumor necrosis factor alpha.

**Figure 2:**
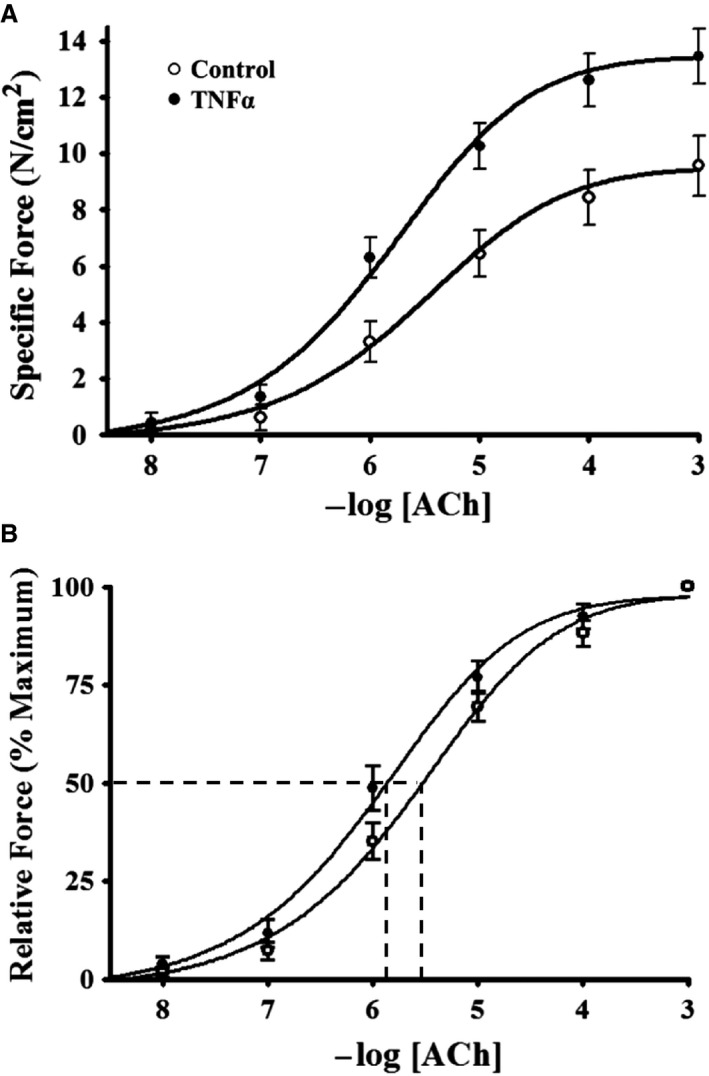
Summary results showing a dependency of porcine ASM‐specific force on ACh concentration (A). After 24‐h TNFα (100 ng/mL) exposure, ASM force increased across all ACh concentrations (A). The sensitivity of force generation to ACh concentrations was assessed by normalizing force to the maximum force (*F*_max_) for each ASM strip (B). The ACh concentration that induced 50% *F*_max_ (EC_50_) was determined as an index of ACh sensitivity. Note that after 24‐h TNFα exposure the EC_50_ shifted leftward compared to control ASM strips. Values are means ± SD. *Significant difference (*P* < 0.05) from control (*n* = 6 animals). ASM, airway smooth muscle; ACh, acetylcholine; TNFα, tumor necrosis factor alpha; SD, standard deviation.

**Figure 3:**
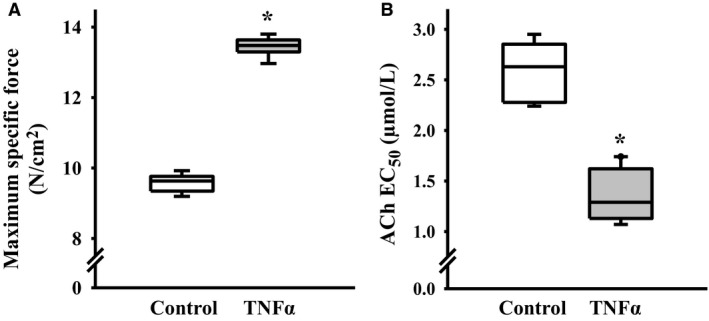
Summary of results showing that 24‐h TNFα (100 ng/mL) exposure increased maximum specific force (*F*_max_) evoked by ACh stimulation (A). Exposure to TNFα (100 ng/mL for 24 h) increased sensitivity to ACh as reflected by a decrease in the ACh concentration that induced 50% *F*_max_ (EC_50_) (B). Values are medians – IQR *Significant difference (*P* < 0.05) from control (*n* = 6 animals). TNFα, tumor necrosis factor alpha; ACh, acetylcholine; IQR, interquartile range.

Isometric force in control and TNFα‐treated ASM strips increased in response to increasing concentrations of ACh stimulation (10 nmol/L–1 mmol/L) (Fig. 1). At each ACh concentration, isometric force was significantly higher in TNFα‐treated ASM strips compared to controls (*P* < 0.05, *n* = 6; Figs. 1, 2A). Maximum specific force (*F*_max_) was obtained at 1 mmol/L ACh in both control and TNFα‐treated groups (Figs. 1, 2A). In TNFα‐treated ASM strips, *F*_max_ increased by ~48% compared to control ASM strips (*P* = 0.001, *n* = 6; Fig. 3A). Force was normalized to *F*_max_ to determine the EC_50_ for ACh, which was ~twofold lower in TNFα‐treated ASM strips compared controls (*P* = 0.001, *n* = 6; Figs. 2B, 3B).

### Effect of TNFα on simultaneous [Ca^2+^]_cyt_ and force responses to ACh stimulation

**Figure 4:**
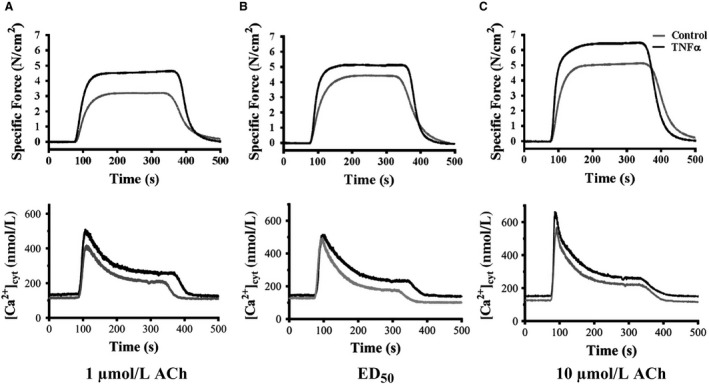
Representative tracings of ACh‐induced elevation of [Ca^2+^]_cyt_ and increased specific force in control and TNFα‐exposed (100 ng/mL, 24 h) ASM strips at three different ACh concentrations. (A) 1 μmol/L ACh; (B) 1.3 μmol/L ACh for control (EC_50_ ) and 2.6 μmol/L ACh for TNFα (EC_50_ ); (C) ACh 10 μmol/L. ACh, acetylcholine; TNFα, tumor necrosis factor alpha.

**Figure 5:**
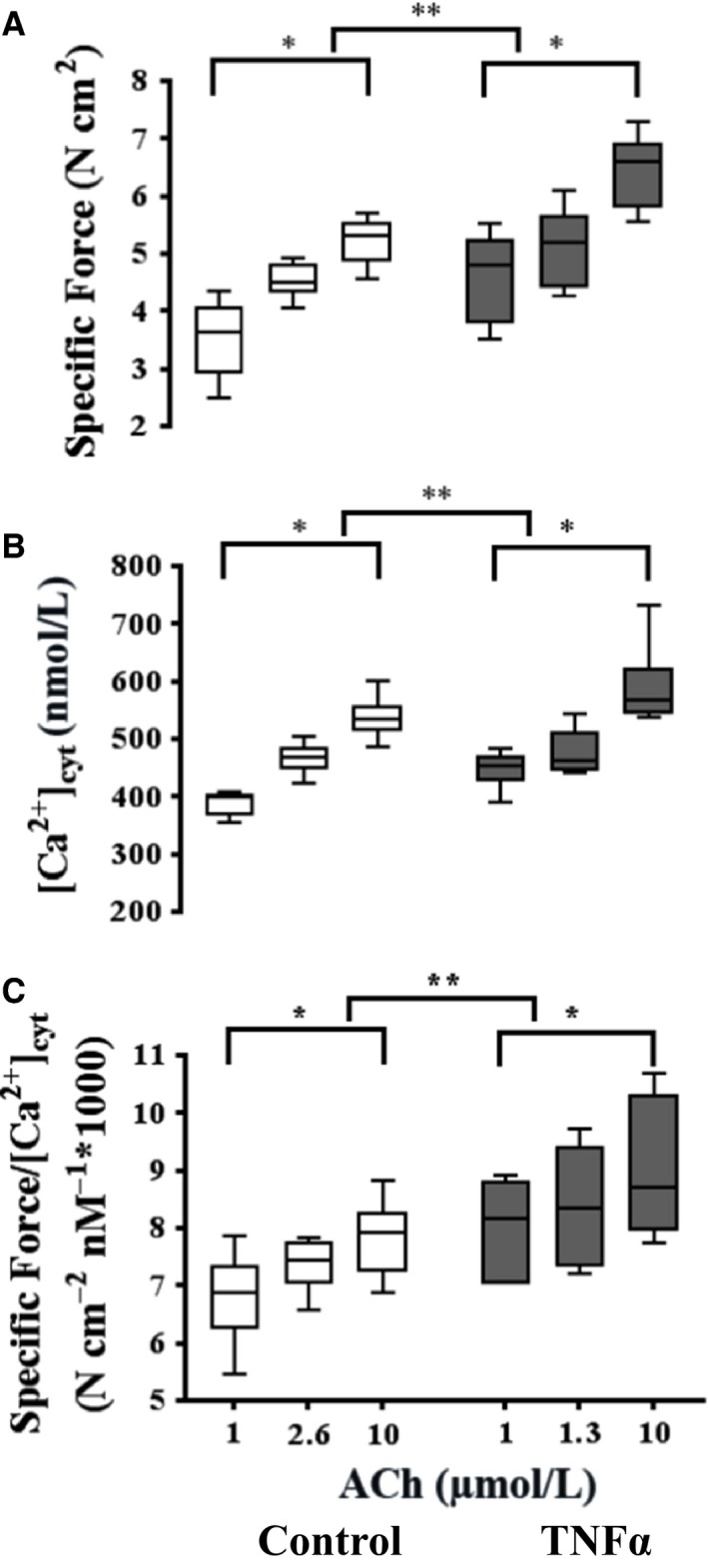
Summary of the specific force (A) and peak [Ca^2+^]_cyt_ (B) responses induced by ACh at three different concentrations (control: 1, 2.6 [EC_50_], and 10 μmol/L; TNF: 1, 1.3 [EC_50_], 10 and 10 μmol/L). Exposure to TNFα (100 ng/mL, 24 h) significantly increased ASM‐specific force across all ACh concentrations (A). TNFα exposure significantly increased the peak amplitude of the ACh‐induced [Ca^2+^]_cyt_ response except at the EC_50_ concentration (B). Due to the disproportionate increase in specific force induced by TNFα exposure, Ca^2+^ sensitivity (force per [Ca^2+^]_cyt_) significantly increased (C). Values are medians – IQR. *Significant difference (*P* < 0.05) versus initial 1 μmol/L ACh for each group (*n* = 6 animals). **Significant interaction between ACh concentration dependence and treatment group. ACh, acetylcholine; TNFα, tumor necrosis factor alpha; ASM, airway smooth muscle; IQR, interquartile range.

The effects of TNF on simultaneous force and [Ca^2+^]_cyt_ responses to ACh stimulation were examined at three different concentrations (1 μmol/L, the EC_50_ [2.6 μmol/L for control and 1.3 μmol/L for TNF], and 10 μmol/L). At each concentration, ACh stimulation induced transient [Ca^2+^]_cyt_ and force responses that were coupled (Fig. 4A4). Overall, the amplitude of the [Ca^2+^]_cyt_ response increased with increasing ACh concentration, and this dose dependency was significantly different between TNFα‐treated and control ASM strips (*P* < 0.05, *n* = 6; Figs. 4, 5B). Stimulation at 1 and 10 μmol/L ACh induced greater amplitude [Ca^2+^]_cyt_ responses in TNFα‐treated ASM strips compared to controls (*P* < 0.05, *n* = 6; Figs. 4A and 4, 5B). However, at the EC_50_ concentration of ACh, the amplitude and time course of the transient [Ca^2+^]_cyt_ responses were comparable between TNFα‐treated and control ASM strips.

In general, ASM force developed rapidly in response to elevated [Ca^2+^]_cyt_ and was maintained for the duration of ACh stimulation (Fig. 4). TNFα treatment significantly increased ASM force responses across the three different ACh concentrations in ASM strips (*P* < 0.01, *n* = 6; Figs. 4, 5A). Given the relatively greater effect of TNFα treatment on ASM force compared to [Ca^2+^]_cyt_ responses, Ca^2+^ sensitivity (force per [Ca^2+^]_cyt_) was increased in TNFα‐treated ASM strips compared to controls across all three ACh concentrations (*P* < 0.05, *n* = 6; Fig. 5C).

### Effect of TNFα on rMLC phosphorylation response to ACh stimulation

**Figure 6:**
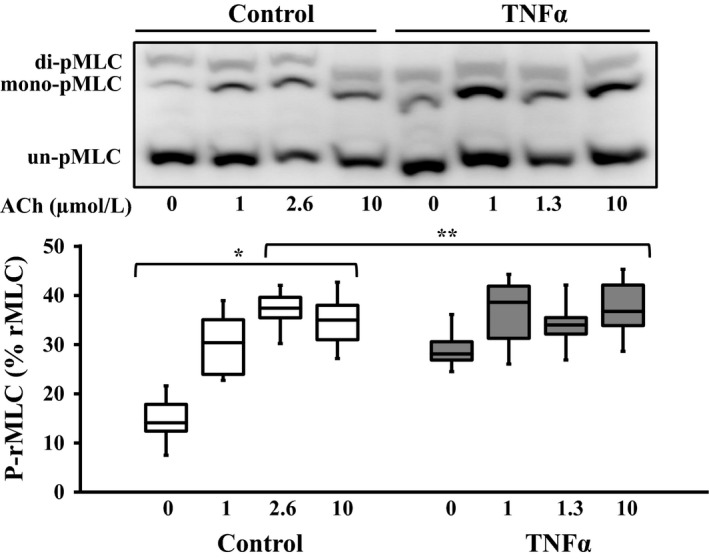
Representative western blots in which phosphorylated (mono‐ and di‐ p‐rMLC) and unphosphorylated (un‐p‐rMLC) rMLC were separated using a modified Phos‐tag™ gels. In control ASM, ACh stimulation increased rMLC phosphorylation (p‐rMLC relative to total rMLC) up to the EC_50_ concentration (2.6 μmol/L) but with no change at 10 μmol/L. Exposure to TNFα (100 ng/mL, 24 h) significantly increased the basal level of rMLC phosphorylation but blunted the ACh‐dependent increase in pMLC. The ratio of p‐rMLC to total rMLC increased with 1 and 2.6 μmol/L ACh stimulation in control ASM strips and phosphorylation of rMLC did not increase at the 10 μmol/L ACh stimulation. In the TNFα‐treated group, basal p‐rMLC was increased in unstimulated ASM strips and there was no significant difference at the other doses of ACh stimulation. Values are medians – IQR. *Significant difference (*P* < 0.05) compared to unstimulated ASM strip within the control group (*n* = 6 animals). **Significant interaction between ACh concentration dependence and treatment group. rMLC, regulatory myosin light chain; ASM, airway smooth muscle; ACh, acetylcholine; TNFα, tumor necrosis factor alpha.

**Figure 7:**
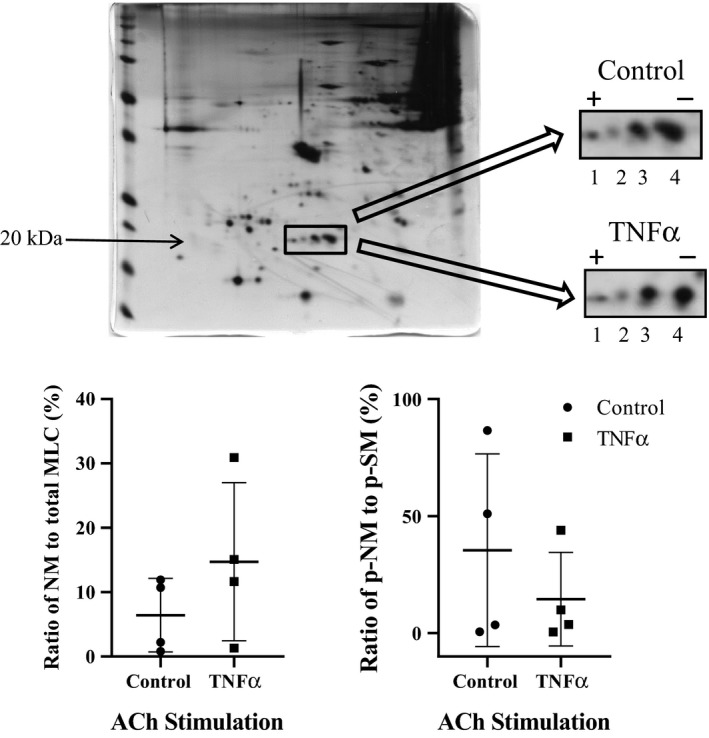
Representative silver‐stained 2‐D gel used for quantifying phosphorylated (dots 1 and 3) and nonphosphorylated (dots 2 and 4) NM and SM rMLC, respectively, in control and TNFα‐treated (100 ng/mL, 24 h) porcine ASM. Exposure to TNFα significantly increased the ratio of NM myosin to total rMLC at the baseline. After ACh stimulation at the EC_50_ concentration (2.6 μmol/L for control and 1.3 for TNFα‐treated ASM), the ratio of phosphorylated NM rMLC to phosphorylated SM rMLC was reduced in TNFα‐treated ASM strips. Values for each of the four animals are presented. NM, nonmuscle; SM, smooth muscle; rMLC, regulatory myosin light chain; TNFα, tumor necrosis factor alpha; ASM, airway smooth muscle; ACh, acetylcholine.

The extent of overall rMLC phosphorylation induced by ACh was examined at three different concentrations (1 μmol/L, the EC_50_ [2.6 μmol/L for control and 1.3 μmol/L for TNFα] and 10 μmol/L). In control ASM, the extent of rMLC phosphorylation increased at 1 and 2.6 μmol/L (EC_50_ for control ASM) ACh stimulation compared to baseline (unstimulated), but did not increase further at 10 μmol/L ACh (*P* = 0.001, *n* = 6; Fig. 6). In TNFα‐treated ASM, the overall extent of rMLC phosphorylation at baseline was elevated compared to controls (*P* = 0.001, *n* = 6; Fig. 6), and there was no dependency of rMLC phosphorylation on the level of ACh stimulation. The overall extent of rMLC phosphorylation in TNFα‐treated ASM was comparable to that observed in control ASM strips during maximal ACh stimulation (Fig. 6). Using 2D gel electrophoresis, we examined the phosphorylated and nonphosphorylated NM and SM myosin at baseline and after stimulation with ACh (at the EC_50_: 2.6 μmol/L for control and 1.3 μmol/L for TNFα). In TNFα‐treated ASM, the ratio of NM myosin to total MLC was slightly increased in unstimulated ASM strips compared to controls (*n* = 4; Fig. 7). After ACh stimulation, the ratio of NM myosin phosphorylation to SM phosphorylation was also slightly decreased in TNFα‐treated ASM strips compared to controls (*n* = 4; Fig. 7).

### Effect of TNFα on MyHC and actin concentrations

**Figure 8:**
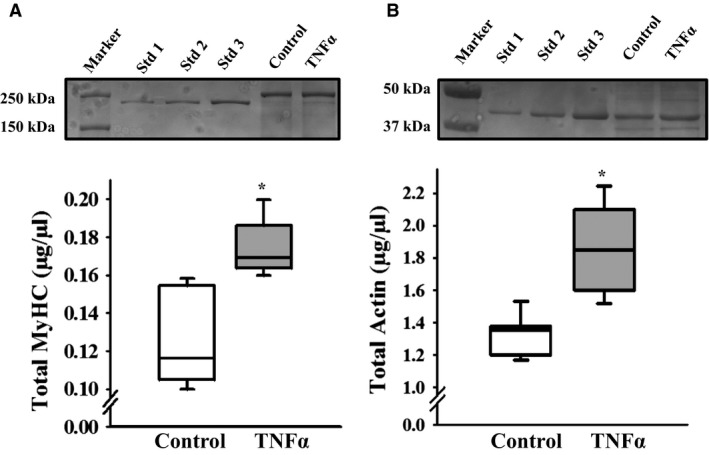
Representative Coomassie‐stained gels used for quantifying MyHC (A) and actin (B) protein concentrations in control and TNFα‐treated (100 ng/mL, 24 h) porcine ASM. Exposure to TNFα significantly increased both MyHC and actin concentration in ASM compared to controls. Values are medians – IQR. *Significant difference (*P* < 0.05) compared to control (*n* = 6 animals). MyHC, myosin heavy chain; TNFα, tumor necrosis factor alpha; ASM, airway smooth muscle; IQR, interquartile range.

In response to 24‐h TNFα treatment, MyHC concentration in ASM increased by ~45% compared to controls (*P* = 0.004, *n* = 6; Fig. 8A). Similarly, actin concentration in TNFα‐treated ASM was ~38% higher than controls (*P* = 0.004, *n* = 6; Fig. 8B).

### Effect of TNFα on actin polymerization

**Figure 9:**
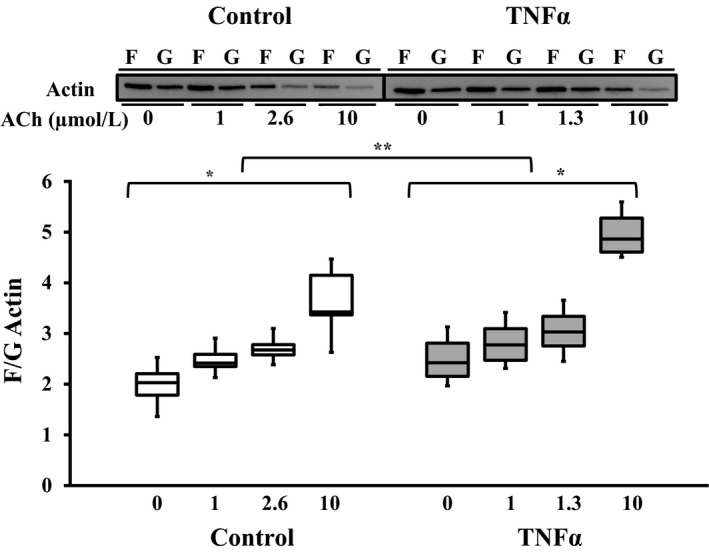
Representative western blots for F‐ and G‐ actin content in ASM strips that were either unstimulated or stimulated by ACh at three different concentrations (control: 1, 2.6 [EC_50_], and 10 μmol/L; TNF: 1, 1.3 [EC_50_], 10 and 10 μmol/L). In both control and TNFα‐treated (100 ng/mL, 24 h) groups, the extent of actin polymerization (increase in F‐ to G‐actin ratio) increased with ACh stimulation. However, exposure to TNFα significantly increased actin polymerization across all ACh concentrations. Values are medians – IQR. *Significant difference (*P* < 0.05) compared to unstimulated ASM strip (*n* = 6 animals). **Significant interaction between ACh concentration dependence and treatment group. ASM, airway smooth muscle; ACh, acetylcholine; TNFα, tumor necrosis factor alpha.

In both control and TNFα‐treated ASM strips, the ratio of F‐ to G‐actin increased significantly with increasing ACh stimulation (*P* < 0.01, *n* = 6; Fig. 9). In TNFα‐treated ASM strips, the F‐ to G‐actin ratio was elevated at baseline (unstimulated) compared to controls (*P* < 0.05, *n* = 6; Fig. 9), and there was greater actin polymerization at maximal ACh stimulation; thus, the dose–response was significantly different between control and TNFα‐treated ASM strips (*P*> 0.05, *n* = 6; Fig. 9). However, at the EC_50_ concentration of ACh stimulation, the F‐ to G‐actin ratio was not significantly different between TNFα‐treated and control ASM.

## Discussion

The results of the present study showed that exposing intact porcine ASM to TNFα for 24 h induces both increased sensitivity to ACh stimulation (hypersensitivity) and an increased force response to ACh (hypercontractility). Hypersensitivity was evidenced by a twofold leftward shift in the EC_50_ for ACh‐induced contractile force responses. Hypercontractility was evidenced by a marked increase in specific force response across all ACh concentrations. Exposure to TNFα enhanced the amplitude of [Ca^2+^]_cyt_ responses to ACh stimulation at 1 and 10 μmol/L; however, at the EC_50_, the amplitude of [Ca^2+^]_cyt_ response to ACh stimulation was comparable between TNFα‐treated and control ASM strips. This suggests that enhancement of the [Ca^2+^]_cyt_ response to ACh does not underlie the hypercontractility induced by TNFα, but is consistent with hypersensitivity. Similarly, TNFα did not enhance the extent of rMLC phosphorylation in response to ACh stimulation. In addition, the dose‐dependent increase in actin polymerization induced by ACh was enhanced by TNFα treatment, but at the EC_50_ ACh concentration, the extent of actin polymerization was comparable. Notably, the increased force response to ACh in TNFα‐treated ASM strips corresponded with increased contractile protein (MyHC and actin) concentrations, reflecting an increased number of contractile units contributing to force.

### Effect of TNFα on ASM sensitivity to ACh

Results of the present study showed that 24‐h TNFα exposure increases the sensitivity of porcine ASM to ACh stimulation. These results are in agreement with previous findings in mouse, rat, and bovine ASM (Kao et al., 1999; Nakatani et al., 2000; Sakai et al., 2004a,2004b). However, in guinea pig trachea, Makwana et al., (2012) reported that TNFα does not alter force responses to MCh stimulation. This apparent discrepancy may relate to the agonist (ACh vs. MCh) tested, the preparation (tracheal rings vs. strips), the experimental condition (TNF exposure time), or species differences. It should also be noted that we performed our experiments at 24°C and cannot exclude possible effects of this lower temperature on our results compared to what may occur at 37°C. In preliminary experiments carried at 37°C, we noted a decreased in ASM force after 24‐h incubation probably due to tissue degradation over time at 37°C. We also noted that Ca^2+^ responses were blunted after 24‐h incubation at 37°C.

In ASM, G‐protein coupled M_3_ muscarinic receptors mediate the effect of ACh on contractile responses by triggering downstream second messenger cascades affecting a transient elevation of [Ca^2+^]_cyt_, increasing rMLC phosphorylation mediating cross‐bridge recruitment and cycling, and cytoskeletal remodeling (Sieck et al., 2001; Gosens et al., 2006; Mitchell et al., 2008; Pera and Penn, 2014). Previous studies have shown that TNFα exposure does not alter the expression of M_3_ muscarinic receptors in ASM but increases Gi and Gq alpha protein expression, which could account for increased ACh sensitivity of force generation (Hakonarson et al., 1996; Hotta et al., 1999; Gosens et al., 2006). In our study, the amplitude of the [Ca^2+^]_cyt_ response, the extent of rMLC phosphorylation, and the extent of actin polymerization in response to the EC_50_ ACh concentration were comparable between TNFα‐treated and control ASM strips, suggesting that the downstream signaling cascades triggered by the G‐protein‐coupled M_3_ muscarinic receptors were not affected by TNFα exposure. On the other hand, basal rMLC phosphorylation was increased in TNFα‐treated ASM strips compared to control ASM strips. This result is in agreement with previous findings in human, rat, and guinea pig ASM (Parris et al., 1999; Hunter et al., 2003; Sakai et al., 2004a,2004b; Goto et al., 2009). An increase in rMLC phosphorylation under resting condition after TNFα exposure could be associated with an increase in resting tension (e.g., phosphorylation of NM rMLC) or muscle tone (phosphorylation of SM rMLC) (Zhang et al., 2015; Chitano et al., 2017). In our study, we did not directly examine the resting tension/tone after TNFα exposure and cannot exclude that TNFα exposure increases ASM strip's resting tension. An increase in rMLC phosphorylation could also reflect an increase in either NM rMLC phosphorylation or SM rMLC phosphorylation (Zhang et al., 2015; Chitano et al., 2017; Zhang and Gunst, 2017). Our results show that NM myosin is slightly increased in TNFα‐treated ASM strips at baseline (unstimulated) compared to controls and phosphorylation of NM myosin could be at least partially responsible for the increase in basal rMLC phosphorylation observed in TNFα‐treated ASM strips.

### Effect of TNFα exposure on hypercontractility

The present study showed that 24‐h TNFα exposure induces ASM hypercontractility, evidenced by a marked increase in specific force responses across a wide range of ACh concentrations. In TNFα‐treated ASM strips, the amplitude of the [Ca^2+^]_cyt_ response to ACh was increased at 1 and 10 μmol/L, but not at the EC_50_ concentration of ACh. We and others have shown that cytokines increase the amplitude of the [Ca^2+^]_cyt_ response to ACh stimulation (White et al., 2006; Sathish et al., 2009; Delmotte et al., 2012), but these effects were not evaluated at the EC_50_ concentration of ACh. The ACh‐induced [Ca^2+^]_cyt_ response reflects the net effect on mechanisms involved in cytosolic Ca^2+^ regulation including ryanodine receptor and IP_3_‐mediated Ca^2+^ release from the sarcoplasmic reticulum (SR), cyclic ADP ribose (CD38)‐mediated enhancement of SR Ca^2+^ release, store‐operated Ca^2+^ entry, sodium–calcium exchanger‐mediated Ca^2+^ influx, sarco/endoplasmic reticulum Ca^2+^‐ATPase, and mitochondrial Ca^2+^ uptake (Kannan et al., 1997; Prakash et al., 1997; Sieck et al., 1997; Prakash et al., 1998; Prakash et al., 2000; Tliba et al., 2003; White et al., 2006; Tirumurugaan et al., 2007; Sieck et al., 2008; Sathish et al., 2009; Sathish et al., 2011a; Delmotte et al., 2012; Jia et al., 2013).

The increase in force after TNFα exposure was much greater than the increase in the amplitude of [Ca^2+^]_cyt_ response, which by definition indicates an increase in Ca^2+^ sensitivity. Sakai and colleagues also found that TNFα exposure increases the Ca^2+^ sensitivity of force generation in response to ACh stimulation in rat ASM (Sakai et al., 2004a,2004b). These investigators attributed the TNFα‐induced increase in Ca^2+^ sensitivity of force generation to an increase in the extent of rMLC phosphorylation through activation of the RhoA/Rho‐kinase pathway, an effect also reported in human and guinea pig ASM after TNFα exposure (Parris et al., 1999; Hunter et al., 2003; Sakai et al., 2004a,2004b; Goto et al., 2009). However, these investigators did not evaluate ACh sensitivity of this response. In the present study, we found that in control ASM strips, increasing ACh concentration increased the extent of rMLC phosphorylation up to the EC_50_ concentration (2.6 μmol/L) with no further change in rMLC phosphorylation at higher ACh concentrations even though force increased. In TNFα‐treated ASM strips, the extent of basal rMLC phosphorylation was already higher and comparable to the maximum extent of rMLC phosphorylation achieved in controls. Importantly, after TNFα treatment, the extent of rMLC phosphorylation was not dependent on ACh concentration.

In the previous studies, we and others have suggested that hypercontractility involves an increase in the number of contractile elements contributing to force via increased actin and MyHC concentrations mediated by polymerization/depolymerization of myosin and actin filaments (Mehta and Gunst, 1999; Jones et al., 1999b; Kuo et al., 2003b; Seow, 2005; Ijpma et al., 2011; Dogan et al., 2017). In permeabilized porcine ASM, we found that the TNFα‐induced increase in maximum Ca^2+^‐activated force was at least partially attributed to an increase in both actin and MyHC concentrations (Dogan et al., 2017). These results are in agreement with other studies that reported a TNFα‐induced increase in actin expression in other cell types (Goldblum et al., 1993; Yokoyama et al., 1997; Wojciak‐Stothard et al., 1998). In permeabilized porcine ASM, the TNFα‐induced increase in MyHC and force was also reflected by an increase in ATP hydrolysis (Dogan et al., 2017). Altogether, our results suggest that 24‐h TNFα exposure induces hypercontractility primarily through an increase in the number of contractile elements contributing to ASM force generation.

Translation of internal cross‐bridge interactions to an external load requires cytoskeletal remodeling and the tethering of contractile elements to the cortical cytoskeleton and cytosolic dense bodies regarded as the anchoring sites for actin filaments to the plasma membrane and within the cytosol, respectively. (Gunst and Tang, 2000; Gunst et al., 2003; Zhang and Gunst, 2008; Zhang et al., 2010; Syyong et al., 2015). Whether TNFα exposure affects the anchoring of actin filament to the cortical cytoskeleton and dense bodies in ASM is unknown. Cytoskeletal remodeling in ASM includes polymerization of G‐ to F‐actin (Mehta and Gunst, 1999; Jones et al., 1999b; Dogan et al., 2017). In the previous studies, we and others reported that ASM force generation is markedly reduced when actin polymerization is disrupted (Mehta and Gunst, 1999; Jones et al., 1999b), which likely reflects a disruption of the tethering of contractile elements to the cortical cytoskeleton. In permeabilized porcine ASM, we reported that the TNFα‐induced increase in force generation was associated with an increase in G‐ to F‐actin polymerization following Ca^2+^ activation (Dogan et al., 2017). However, in a permeabilized preparation, the plasma membrane is obviously disrupted, which certainly could impact cytoskeletal remodeling. In intact porcine ASM, we showed an ACh‐dependent increase in actin polymerization in both control and TNFα‐exposed ASM strips. Interestingly, the baseline F‐ to G‐actin ratio was increased in TNFα‐exposed ASM compared to control ASM. Others studies have reported that IL‐13 also increases G‐ to F‐actin polymerization (Zhang and Gunst, 2008; Chiba et al., 2009; Zhang et al., 2012).

In conclusion, the 24‐h TNFα exposure induces enhancement of ASM force generation to ACh stimulation, due to an increased sensitivity to ACh, increased [Ca^2+^]_cyt_ response to ACh, increased Ca^2+^ sensitivity of ASM force generation, increased basal rMLC phosphorylation, increased number of contractile elements, and increased actin polymerization. However, taken together, it appears that the major factor contributing to TNFα‐induced ASM hypersensitivity to ACh and hypercontractility is an increase in the number of contractile units contributing to force.

## Conflict of Interests

The authors have no conflicts of interests.

## Data Availability

The datasets generated and analyzed in this study are available from the corresponding author on reasonable request.
